# Sex differences in plasma clozapine and norclozapine concentrations in clinical practice and in relation to body mass index and plasma glucose concentrations: a retrospective survey

**DOI:** 10.1186/s12991-015-0075-x

**Published:** 2015-11-14

**Authors:** Simon G. Anderson, Mark Livingston, Lewis Couchman, Daniel J. Smith, Moira Connolly, Joan Miller, Robert J. Flanagan, A. H. Heald

**Affiliations:** Institute of Cardiovascular Sciences, University of Manchester, Manchester, UK; The George Institute for Global Health, Nuffield Department of Population Health, Oxford Martin School, University of Oxford, Oxford, UK; Department of Blood Sciences, Walsall Manor Hospital, Walsall, UK; Toxicology Unit, Department of Clinical Biochemistry, King’s College Hospital NHS Foundation Trust, London, UK; Institute of Health and Wellbeing, University of Glasgow, Glasgow, UK; Intensive Psychiatric Care Unit, Gartnavel Royal Hospital, Glasgow, UK; Pharmacy, Prestwich Hospital, Prestwich, Greater Manchester, UK; Department of Medicine, Leighton Hospital, Crewe, CW1 4QJ UK; School of Medicine and Manchester Academic Health Sciences Centre, University of Manchester, Manchester, UK

**Keywords:** Clozapine, Gender, BMI, Glucose

## Abstract

**Background:**

Clozapine is widely prescribed and, although effective, can cause weight gain and dysglycemia. The dysmetabolic effects of clozapine are thought to be more prevalent in women with this gender on average attaining 17 % higher plasma clozapine concentrations than men.

**Methods:**

We investigated the relationship between dose, body mass index (BMI), plasma glucose concentration, and plasma clozapine and *N*-desmethylclozapine (norclozapine) concentrations in 100 individuals with a severe enduring mental illness.

**Results:**

Mean (10th/90th percentile) plasma clozapine concentrations were higher for women [0.49 (0.27–0.79) mg/L] compared with men [0.44 (0.26–0.70) mg/L] (*F* = 2.2; *p* = 0.035). There was no significant gender difference in the prescribed clozapine dose. BMI was significantly higher in women [mean (95 % CI) = 34.5 (26.0–45.3)] for females compared with 32.5 (25.2–41.0) for males. Overall, BMI increased by 0.7 kg/m^2^ over a mean follow-up period of 210 days. A lower proportion, 41 % of women had a fasting blood glucose ≤6.0 mmol/L (<6.0 mmol/L is defined by the International Diabetes Federation as normal glucose handling), compared with 88 % of men (*χ*^2^ = 18.6, *p* < 0.0001).

**Conclusions:**

We have shown that mean BMI and blood glucose concentrations are higher in women prescribed clozapine than in men. Women also tended to attain higher plasma clozapine concentrations than men. The higher BMI and blood glucose in women may relate to higher tissue exposure to clozapine, as a consequence of sex differences in drug metabolism.

## Background

Clozapine is widely prescribed for treatment-resistant schizophrenia (TRS) and is also indicated in bipolar I disorder. Evidence of superior efficacy compared with other first and second generation neuroleptics used for the treatment of psychosis is manifest in individual trials and in meta-analyses [1]. However, there is concern because the common longer-term side effects of clozapine include weight gain, elevation of blood glucose concentrations, dyslipidemia (and ‘metabolic syndrome’), as well as somnolence, dizziness, hypersalivation, gastrointestinal hypomotility, peripheral oedema, and postural hypotension.

The dysmetabolic effects of clozapine are thought to be more prevalent in women [2] and it has been shown that gender does have an influence on the observed inter-patient variability in plasma clozapine concentrations, with women on average attaining 17 % higher plasma clozapine concentrations than men at constant dose irrespective of smoking habit [3]. This is in keeping with already identified gender-related differences in pharmacokinetics for some drugs, including theophylline and several benzodiazepines, known since the 1980s [4].

The initial clozapine dose is 12.5 mg/day, increasing gradually to 300–400 mg/day, up to a licensed maximum of 900 mg/day.

Therapeutic drug monitoring (TDM) of clozapine and *N*-desmethylclozapine (norclozapine) is useful when assessing therapeutic effectiveness and for dose optimisation [5, 6] and may also be relevant to assessing the metabolic consequences of treatment with clozapine.

In order to gather information as to whether the gender differences in clozapine and norclozapine concentrations might influence body mass index (BMI), glycaemia and lipidemia, we have examined data from a single tertiary UK hospital clozapine clinic.

## Methods

We carried out a retrospective review of inpatients and outpatients (*n* = 100) with clozapine TDM and consecutively assessed results in 2014. All patients had schizophrenia or schizoaffective disorder. All patients were treatment resistant, hence treatment with Clozapine. Out of the 100 patients, 82 were diagnosed with Schizophrenia and 18 with Schizoaffective Disorder.

Data was obtained from the analysis of plasma samples submitted for routine TDM in patients seen in Salford Community Mental Health Centres or patients seen in clozapine clinics on the Prestwich Hospital site (Prestwich, Greater Manchester, UK). Ethical approval was obtained for the study.

Data recorded with each sample included: prescribed clozapine dose (mg/day), duration of clozapine treatment, age, gender, body weight (kg), height (m), smoking habit (number of cigarettes/day), and any other information that could aid interpretation of the results, for example, co-prescribed drugs (and doses).

Plasma clozapine concentrations were related to BMI (at time of sampling) and plasma glucose concentrations (at the time of sampling). For clozapine TDM, it was requested that samples be collected at least 6 h post-dose (‘trough’ samples). BMI was calculated as weight (kg)/height^2^ (m^2^).

Plasma glucose and lipids were analyzed using a Roche Cobas 8000 autoanalyser, (Roche Diagnostics, Burgess Hill, West Sussex, UK), according to the manufacturer’s instructions. Analysis was carried out at the biochemistry laboratory at Salford Royal Hospital, Greater Manchester, UK. Plasma clozapine and norclozapine were measured at King’s College Hospital (London, UK) by high-performance liquid chromatography with ultraviolet detection (240 nm) after extraction into methyl *tert*-butyl ether at pH 10.6. Intra-assay precision was between 3.8 and 6.6 %. Corresponding inter-assay precision was between 5.6 and 15.2 % [7].

### Statistical analysis

All analyses were conducted using Stata/MP statistical software (version 13.1, College Station, Texas, USA). Categorical variables were compared using the Chi-squared test and continuous variables were compared using *t* tests or analysis of variance. Distributions of BMI and clozapine by gender were compared using Epanechnikov kernel density plots. The ethnicity profile of men and women was similar.

## Results

A total of 41 female and 59 male patients were included in the study. Males were on average younger than females with a mean [95 % confidence interval (CI)] age of 36.9 (33.9–39.8) years and 39 (35.4–42.7) years, respectively. The duration of clozapine treatment was not significantly different (*p* = 0.6), with a mean (95 % CI) duration of 4.4 (1.2–10.3) years in males and 5.1 (2.3–7.9) years in females, and there was no significant difference in prescribed clozapine dose, with a mean (95 % CI) dose of 433 (389–477) mg/day and 425 (388–462) mg/day for males and females, respectively. Where smoking status was recorded, 75.5 % of males and 63.9 % of females smoked.

Overall, BMI increased by 0.7 kg/m^2^ over a mean follow-up period of 210 days from the start of weight monitoring.

Twelve patients were taking a second neuroleptic agent [aripiprazole (*n* = 6), amisulpride (*n* = 4), and haloperidol (*n* = 2)]. Of the females, five were prescribed an oestrogen-containing oral contraceptive pill and three were prescribed an oestrogen-containing hormone replacement treatment.

### Clozapine and norclozapine TDM

**Fig. 1:**
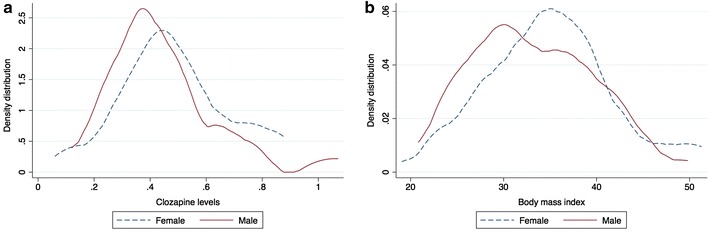
Kernel density distribution of age-adjusted **a** clozapine levels and **b** BMI by gender

Mean (10th/90th percentile) plasma clozapine concentrations were higher for females [0.49 (0.27–0.79) mg/L] compared with males [0.44 (0.26–0.70) mg/L] (*F* = 2.2; *p* = 0.035, Fig. 1a). There was no gender difference in plasma norclozapine concentrations [0.31 (0.26–0.35) mg/L and 0.31 (0.27–0.34) mg/L for males and females, respectively]. A higher proportion of the samples from females had plasma clozapine concentrations of 0.60–1.00 mg/L (25 % of samples from females compared with 14 % of samples from males) and >1.00 mg/L (11 % of samples from females compared with 6.6 % of samples from males).

### Metabolic investigations

BMI was significantly higher in women [mean (95 % CI) = 34.5 (26.0–45.3)] for females compared with 32.5 (25.2–41.0) for males; *F* = 3.8, *p* = 0.02, Fig. 1b). For women, 41 % had a fasting blood glucose ≤6.0 mmol/L (<6.0 mmol/L is defined by the International Diabetes Federation as normal glucose handling [8]), compared with 88 % of men (*χ*^2^ = 18.6, *p* < 0.0001). High-density lipoprotein (HDL)-cholesterol was significantly lower in males than in females [mean (95 % CI) = 1.1 (0.97–1.4) mmol/L and 1.2 (1.1–1.3) mmol/L for males and females, respectively; *F* = 4.3; *p* = 0.04].

## Discussion

These data provide further evidence that females are attaining higher plasma clozapine concentrations in routine clinical practice than males after allowing for differences in prescribed dose and smoking status [9]. The effect of gender on plasma clozapine concentration might at first sight be considered anomalous since females tend to have more adipose tissue than males; hence, lipophilic drugs such as clozapine might be expected to show a lower plasma concentration at a given dose than males. However, this anomaly may be explained by evidence that (1) men have higher activities of drug-metabolizing cytochromes than women and (2) there are known sex differences in pharmacokinetic factors, in particular, renal clearance rate is generally slower in females [2]. Another potential factor, which might explain the gender difference in clozapine levels and the clozapine:norclozapine ratio, is that women are probably more likely to be fully adherent with their medication than men.

In this study, there was no significant gender difference associated with plasma norclozapine concentrations, and so the clozapine:norclozapine ratio was higher in females. This is an important finding, consistent with these known sex differences in drug metabolism and clearance, since females may be at increased risk of clozapine accumulation than males at a given dose.

We have also shown that BMI and plasma glucose concentrations were on average higher in the females than in the males in this study. These findings may relate directly to greater tissue exposure to clozapine and/or norclozapine. Differences by gender in HDL-cholesterol (lower in men) were in keeping with general population patterns.

Whilst our data are based on observational findings in a relatively small number of samples, and take no account of possible partial- or non-adherence to medication, the higher clozapine concentrations and the higher clozapine:norclozapine ratio may be significant factors determining the increased incidence of weight gain and dysmetabolic profiles in females prescribed clozapine. They may also explain increased incidence of other clozapine-related side effects in females. A potential factor that requires further investigation is the effect of oestrogen and oral contraceptives in inhibiting drug-metabolizing enzyme activity in women [10].

## Conclusions

Though treatment with clozapine offers significant benefits for many patients with TRS, it is associated with a high incidence of substantial weight gain, especially in females. We have shown that mean BMI and blood glucose concentrations are higher in females prescribed clozapine than in males, and provided further evidence from TDM data that this may relate to higher tissue exposure over time to clozapine and/or norclozapine.

It is clear that plasma clozapine concentrations well above the suggested (sex-independent) target ranges are more frequent in females. Psychiatrists should take into account gender differences when prescribing clozapine, and should make use of TDM to guard against clozapine accumulation, which may be more likely in females prescribed the drug, with the attendant metabolic consequences.

## Abbreviations

BMI: body mass index
CI: confidence interval
HDL: high-density lipoprotein
TDM: therapeutic drug management
TRS: treatment-resistant schizophrenia
